# A movable unshielded magnetocardiography system

**DOI:** 10.1126/sciadv.adg1746

**Published:** 2023-03-29

**Authors:** Wei Xiao, Chenxi Sun, Liang Shen, Yulong Feng, Meng Liu, Yulong Wu, Xiyu Liu, Teng Wu, Xiang Peng, Hong Guo

**Affiliations:** State Key Laboratory of Advanced Optical Communication Systems and Networks, School of Electronics, and Center for Quantum Information Technology, Peking University, Beijing 100871, China.

## Abstract

Magnetocardiography (MCG), which uses high-sensitivity magnetometers to record magnetic field signals generated by electrical activity in the heart, is a noninvasive method for evaluating heart diseases such as arrhythmia and ischemia. The MCG measurements usually require the participant keeping still in a magnetically shielded room due to the immovable sensor and noisy external environments. These requirements limit MCG applications, such as exercise MCG tests and long-term MCG observations, which are useful for early detections of heart diseases. Here, we introduce a movable MCG system that can clearly record MCG signals of freely behaving participants in an unshielded environment. On the basis of optically pumped magnetometers with a sensitivity of 140 fT/Hz^1/2^, we successfully demonstrated the resting MCG and the exercise MCG tests. Our method is promising to realize a practical movable multichannel unshielded MCG system that nearly sets no limits to participants and brings another kind of insight into the medical diagnosis of heart disease.

## Teaser

A movable unshielded magnetocardiography system is developed to record cardiac magnetic signals of moving participants.

## INTRODUCTION

Magnetocardiography (MCG) is a noninvasive method for the assessment of various heart diseases, which records the biomagnetic signals generated from the heart using high-sensitivity magnetometers. Because of the homogeneous permeability of the body (*1*), the cardiac magnetic signals can travel through the body with almost no disturbance, which makes MCG a potential advanced diagnostic modality for cardiac diagnosis compared with the traditional electrocardiography (ECG) that measures the cardiac electric signals. Thus, MCG is expected to be more sensitive and can be especially helpful for early diagnosis of numerous heart diseases (*2*–*6*) and monitoring the fetal heart health (*7*–*9*).

The resting ECG test, which records the ECG signal of participants in rest, is typically taken as part of the routine checkups for people in clinic applications (*10*–*11*). However, some heart abnormalities can be seen only during exercise (*12*), such as the common unexplained chest pain. The exercise ECG (eECG), which records the electrical signal of patients in exercise, is a widely used diagnostic procedure in the evaluation of both normal participants and cardiologic patients with coronary heart disease (*13*–*16*).

Because the MCG is more sensitive to tangential cardiac currents and circular vortex currents and the ECG is more sensitive to the radial currents in principle (*1*, *17*, *18*), it is believed that the exercise MCG (eMCG) contains information complementary to the eECG (*19*, *20*) and shows a better sensitivity to the deviations from the normal activation direction (*21*, *22*).

Besides, compared to the eECG requiring a direct contact between the electrodes and the skin, the eMCG is noncontact, which is a crucial advantage for patients with skin burns and can avoid the noise caused by the unstable contact between the sensors and the skin during exercise. Therefore, a movable MCG system that can be used as conveniently as the ECG system is highly demanded for cardiac diagnoses, such as eMCG tests and long-term MCG observations.

However, despite the great clinical significance of movable MCG systems, few studies have been reported (*14*, *15*, *23*–*27*). The MCG signal is about seven orders of magnitudes weaker than the Earth’s magnetic field, which requires a high-sensitivity magnetometer and an ability to extract such a faint magnetic field signal from the noisy environment. Superconducting quantum interference devices (SQUIDs) are one of the most sensitive devices and the mainly used detectors of choice for cardiac magnetic field measurements now. However, the traditional SQUID-based MCG system requires a large Dewar for the cryogenic operational condition of the sensor, which restricts the activities of participants. Besides, the MCG system is often operated in a magnetically shielded room (MSR) for suppressing the ambient magnetic field noise. Because of the expensive SQUID sensors, bulky Dewar, and high-cost MSR, the SQUID-based MCG system is mainly used in laboratories for research purposes rather than widely used in hospitals for clinical applications.

As a rapidly expanding field of medical research, the community of cardiac magnetism needs the development of alternative, low-cost, movable, and unshielded MCG signal detectors, which would make a wider distribution and application of magnetic diagnostics. Many efforts have been made to develop such kinds of magnetometers, such as the induction coil magnetometer (*28*), fluxgate magnetometers (*29*), and magnetoresistive magnetometers (*30*, *31*). However, these magnetometers used for biomagnetic measurements are either not sensitive enough, especially for low-frequency field measurements on the order of hertz, to give a clean MCG signal or need large magnetic field–sensitive elements that are hard to be miniaturized for multichannel MCG measurements.

The emerging optically pumped magnetometer (OPM), which is based on the Zeeman effect and light-atom interactions, is one of the most sensitive magnetometers that can reach a sensitivity as high as the SQUID sensor (*32*–*34*) without the cryogenic operational condition. With the advantages of OPMs, such as noncryogenic, small size, movable, custom-design friendly, and less expensive, the OPM is a promising sensor for many kinds of biomagnetic field measurements. Therefore, it is possible to construct a movable unshielded MCG system with OPMs. Previously, biomagnetic signal measurements based on OPMs are often performed in magnetically shielded environments (*35*, *36*) due to the magnetic field noise from surroundings. Despite there being several works on unshielded biomagnetic signal measurements (*35*, *37*), these shielded or unshielded measurements normally require the participant to remain still during the measurement. These requirements for the magnetic field environment and participants further limit the potential advantages of OPM-based MCG in clinical applications, such as eMCG tests and long-term MCG observations.

Here, we develop an unshielded OPM-based movable MCG system that allows the participant to move around freely in the natural Earth’s field. With the MCG system, we demonstrate the measurement of resting MCG and eMCG signals. The OPM used in the MCG system is based on a self-oscillating frequency tracking technique (see details in Materials and Methods), which is different from the conventionally used spin-exchange relaxation-free magnetometer (*38*, *39*). The self-oscillating magnetometer could cover the Earth’s field (*40*–*42*) rather than can only be operated in near-zero magnetic fields.

To distinguish the faint eMCG signal of only a few picotesla to tens of picotesla from the Earth’s field (about 54,500 nT in Beijing, China), the magnetometers not only have to be sensitive enough to detect the MCG signal but also should be robust enough to work normally in a moving platform, i.e., the freely behaving participants. In our movable MCG system, two robust self-oscillating OPMs are used to perform gradiometric detection for reducing the magnetic field fluctuations. Under the unshielded Earth’s field (about 54,500 nT in Beijing, China), the homemade magnetic gradiometer has a magnetic field gradient noise floor (sensitivity) of ∼200 fT_rms_/Hz^1*/*2^ (1 fT = 10^−15^ tesla) with a 15-cm baseline, which indicates a sensitivity of ∼140 fT_rms_/Hz^1*/*2^ for each OPM. Furthermore, the OPMs feature a low heading error (*43*) of less than ±0.5 nT, which is an important noise for magnetic measurement in a moving platform where the relative direction of the sensor to the magnetic field is variable. We also characterize the technical issues to be solved and the corresponding improvements to be implemented for realizing further sensitive and miniaturized multichannel sensors.

## RESULTS

In this section, we describe the measurement results of our movable MCG system in unshielded Earth’s field. First, we briefly introduce the movable unshielded MCG system. Second, we present the MCG measurements in two typical conditions. One is the resting MCG measurements, in which the participants sit on a chair in rest. The other is the eMCG measurements, in which the participants stand on the ground and do the high-knee exercise during recordings. All the experiments are performed under the approval of the local Institutional Review Board, and all the participants provided written informed consent.

### Unshielded MCG system in a moving platform

To make the MCG system work well and have a good performance in an unshielded moving platform, i.e., a freely behaving participant, we need to solve two key problems. The first problem is that the vibration of the moving platform may cause the OPMs stop working or have a poor performance due to unstable sensor structure, vibration-sensitive lasers, etc. The second problem is that the magnetic field noise from the surroundings will greatly increase the noise floor of the OPMs.

**Fig. 1.**
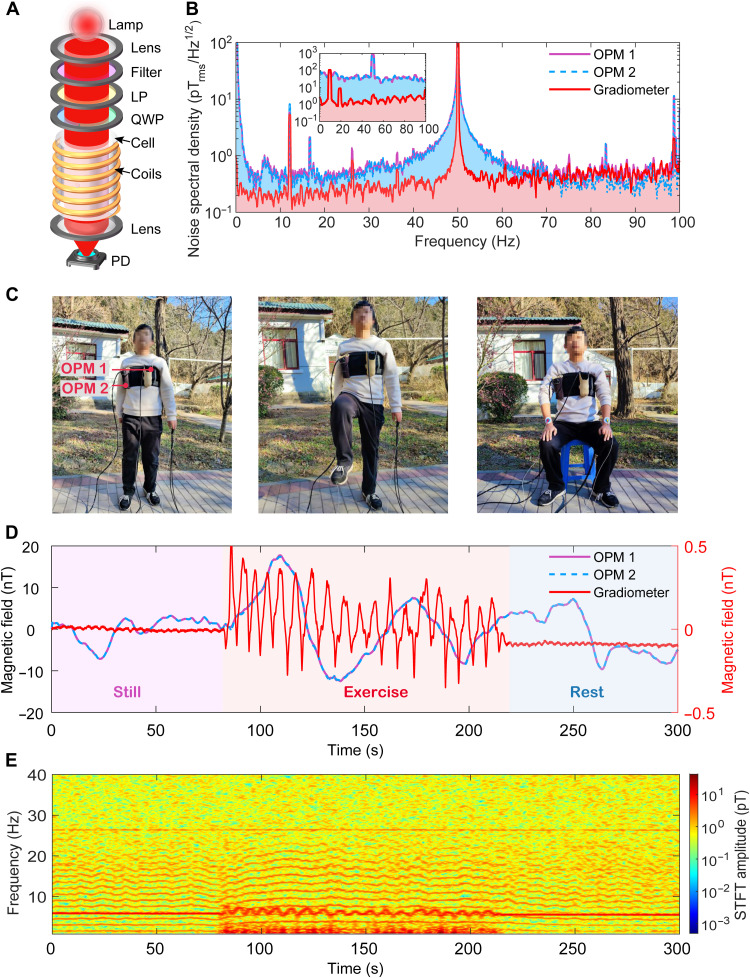
Movable unshielded MCG system using OPMs. (**A**) Schematic of the OPM. LP, linear polarizer; QWP, quarter-wave plate; PD, photodiode. (**B**) The noise floor of the MCG system in the natural open environment. The inset shows the noise floor of the two OPMs and the gradiometer in a noisy outdoor environment created by magnetic coils, indicating the ability of the unshielded MCG system to reduce the common-mode magnetic field noise. (**C**) The overall arrangement of the practical unshielded MCG system. One OPM sensor is placed right on the left chest of participants and the other OPM sensor is placed far away (~15 cm) from the heart. (**D**) Measured motion artifacts in an eMCG test. The artifacts caused by the motion of OPMs in gradient geomagnetic fields are greatly suppressed by operating OPMs in the gradiometric mode. (**E**) Time-frequency spectrogram of MCG signals recorded with the gradiometer by using the short-time Fourier transform (STFT).

The schematic of the OPM is shown in Fig. 1A. Light from the lamp is collected by a lens and sent through optical elements to produce circularly polarized light for both pumping and probing the magnetically sensitive atoms in the vapor cell (see details in Materials and Methods). To make sure that the OPMs work normally in a moving platform, all components in the miniaturized OPMs are fixed firmly by a well-designed structure and have been further glued together. Besides, the light source of the OPM is a cesium lamp with temperature stabilization rather than the commonly used diode laser. Benefiting from the stabilized output power and large linewidth of the cesium lamp, the OPM is expected to be less sensitive to the changes in the environment, such as temperature and vibrations.

For MCG system that is operated in an unshielded environment, the common-mode noise rejection ratio (CMRR), which suggests the ability of suppressing the common-mode magnetic noise, plays an important role in determining the performance of the magnetic gradiometer. A high CMRR indicates that the gradiometer could work in a noisy environment. To achieve a higher CMRR with the self-oscillating OPMs, a frequency estimation program to precisely estimate the Larmor frequency is developed (see details in Materials and Methods).

Figure 1B shows the performances of the OPMs and gradiometer in a natural open environment. We achieve a sensitivity of ∼140 fT_rms_/Hz^1*/*2^ for each OPM and a sensitivity of ∼200 fT_rms_/Hz^1*/*2^ for the gradiometer. These frequency spikes in the noise spectral density are mainly caused by the external magnetic disturbance of electronic devices and the cross-talk of driving magnetic fields in OPMs. To characterize the ability to reject the common-mode noise, we apply magnetic white noise to the OPMs with a large magnetic field coil in an unshielded environment, and the noise spectral density of the two OPMs and the gradiometer is shown in the inset of Fig. 1B. The measured CMRR of the gradiometer is about 80 at 1 Hz. The decrease of the CMRR at higher frequencies mainly results from the nonidentical frequency responses of the two OPMs. The measured frequency responses of the two OPMs are almost identical with a −3-dB bandwidth of ∼180 Hz (see details in the Supplementary Materials).

As shown in Fig. 1C, to fix the OPM sensors on the chest of participants, we make a vest with two pockets. The measurement OPM sensor is placed in the pocket that is closer to the heart of participants and the reference OPM sensor is far away (∼15 cm) from the heart. Because the environment magnetic noise is taken as the common-mode noise for the two sensors and the amplitude of MCG signal detected by the reference OPM sensor is much weaker (>1 order) than that of the measurement OPM sensor for the longer measuring distance between the measurement sensor and the heart, we can enhance the signal-to-noise ratio (SNR) of MCG signal by subtracting the measurement results of the two OPM sensors. In this case, the two OPM sensors are operated in a gradiometric mode, where the common-mode noise is suppressed but most of the MCG signal is retained.

Figure 1D shows a measurement of motion artifacts with the OPMs in an eMCG test. The participant wearing the MCG system is asked to stand still for 80 s, exercise (high-knee walking) for 140 s, and then rest for 80 s. All the MCG signals are recorded with participants facing east for larger signal amplitudes (see details in Materials and Methods). The purple line and the blue line show the measured magnetic fields of the two OPMs, respectively. The red line shows the subtraction of the measurements of the two OPMs. To clearly show the low-frequency artifacts caused by motion, the data in Fig. 1D are filtered with a 10-Hz low-pass filter and subtracted from the field offset. It can be seen in Fig. 1 (D and E) that the OPMs can work normally during the eMCG measurement, and there are no spikes that could be caused by unstable optical elements, unstable electrical connections, poor working conditions of the light source, etc. When the participants stand still or rest, breathing artifacts (about 20 pT) with a period of ∼3 s in the gradiometer can be observed. For the exercise process, the motion artifacts caused by the vigorous exercise are relatively large, which is up to about ∼30 nT. However, most of the common-mode motion artifacts are suppressed in the gradiometer, and the motion artifacts left are less than 1 nT. Considering the magnetic field gradient of ∼0.15 nT/cm in the experimental environment, we can roughly infer that there is about 2-m movement of the participant during the exercise, which agrees with the observed movements. The motion artifacts are reasonable and can be further suppressed with a higher-order magnetic gradiometer.

### Resting MCG signal

To test our movable MCG system in an unshielded Earth’s field, we first conduct a resting MCG signal measurement in the yard of our laboratory. During the recordings, the participant keeps away (∼6 m) from the houses that contain many working electronic devices to avoid the magnetic disturbance and the large magnetic field gradient. participants need not take off their clothes to attach the sensor on the skin, which is different from the ECG measurement, but must remove the magnetic materials, such as keys, phones, and coins, to reduce the magnetic interference to the MCG system.

**Fig. 2.**
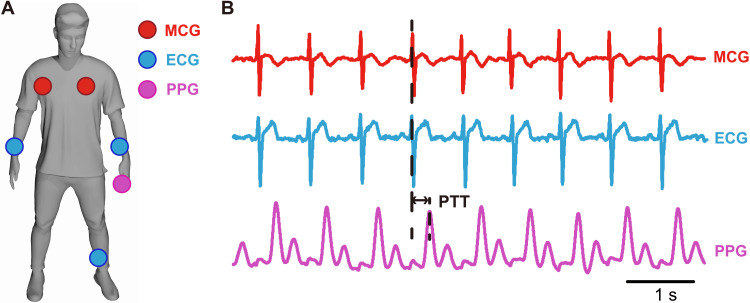
Resting MCG measurement results. (**A**) Arrangement of the MCG, ECG, and PPG (photoplethysmography) sensors when recording cardiac signals. (**B**) Cardiac signals recorded with the MCG, ECG, and PPG sensors. The pulse transit time (PTT) is the time delay between the PPG and MCG (ECG) signal for the same cardiac cycle.

During the recording of resting MCG signals, the participant wearing the MCG system is asked to sit on a plastic chair in rest for 300 s. Besides the MCG recordings, two more methods are also used to synchronously monitor the cardiac activities. We measure the head II ECG signal as the blue circles shown in Fig. 2A and also monitor the photoplethysmography (PPG) signal, with the sensor fixed at the finger of participants, as the purple circle shown in Fig. 2A. PPG is an optical technique to detect blood volume changes in the microvascular bed of tissue by measuring the variation of light absorption. In our experiment, we measure the magnetic cardiac signals of resting healthy participants in the unshielded Earth’s field to test the performance of our MCG system and compare the MCG result with ECG and PPG.

Figure 2B shows a part of the measurement result of the cardiac activity recordings. Typically, there are three main components to an MCG or ECG signal: the P-wave, which represents the depolarization of the atria; the QRS complex, which represents the depolarization of the ventricles; and the T-wave, which represents the repolarization of the ventricles. There is a clear period in the amplitude of the magnetic field signal every ∼0.7 s, the same as that of ECG and PPG. It is clear to see the QRS and T-waves in the MCG signals without any signal averaging.

Because the MCG and ECG signal origins from the same cardiac activities, there is almost no time delay between the two signals. However, because the PPG sensor is worn on the finger of participants, there is a time delay between the PPG and MCG (ECG) signal for the same cardiac cycle, which is called the pulse transit time (PTT). Because the pulse pressure waveform is generated by the ejection of blood from the left ventricle, it is usually calculated as the time between the R-peak of MCG (ECG) signal and a reference point on the plethysmograph. As shown in Fig. 2B, the PTT is estimated to be about 280 ms when it is defined as the time delay between the R-peaks of MCG (ECG) signals and the maximum points of the PPG periods. Because the measurement result of the resting MCG signal, shown in Fig. 2B, matches well with the ECG and PPG signal, our movable MCG system is proved to be capable of observing the resting MCG signal of participants in an unshielded environment.

### Exercise MCG signal

With the wearable OPM, the eMCG signal is further measured to demonstrate the feasibility of detecting MCG signals of freely behaving participants in an unshielded Earth’s field. Similar to the eECG, eMCG records the heart’s response to the stress of exercise.

We measure the eMCG signals for three healthy male individuals aged between 22 and 25 years old. During the measurement, the participants wearing our MCG system (see Fig. 1C) stand in the yard and keep away (∼6 m) from the houses. In the experiment, the participants are asked to perform three tasks: stand still (S stage) for 80 s, do the high-knee exercise as a cardio-intensive exercise for 140 s, and then stand rest for 80 s. During the exercise stage, the participants raise one knee at a time as high as their hips and then switch to the opposite knee. At the same time, the participants hold their hands in front of their abdomen. Considering a cardiac cycle typically ranges from 0.4 to 1 s for a healthy participant, the participants do the high-knee exercise in slow time to avoid mixing the frequency band of motion artifacts and eMCG signals. Figure 1 (D and E) shows the motion artifacts during the eMCG test, and the periodic artifacts with an amplitude of ∼1 nT can be clearly observed during high-knee exercise. There are about 20 periods during the exercise, and each period corresponds to one left knee raising and one right knee raising.

**Fig. 3.**
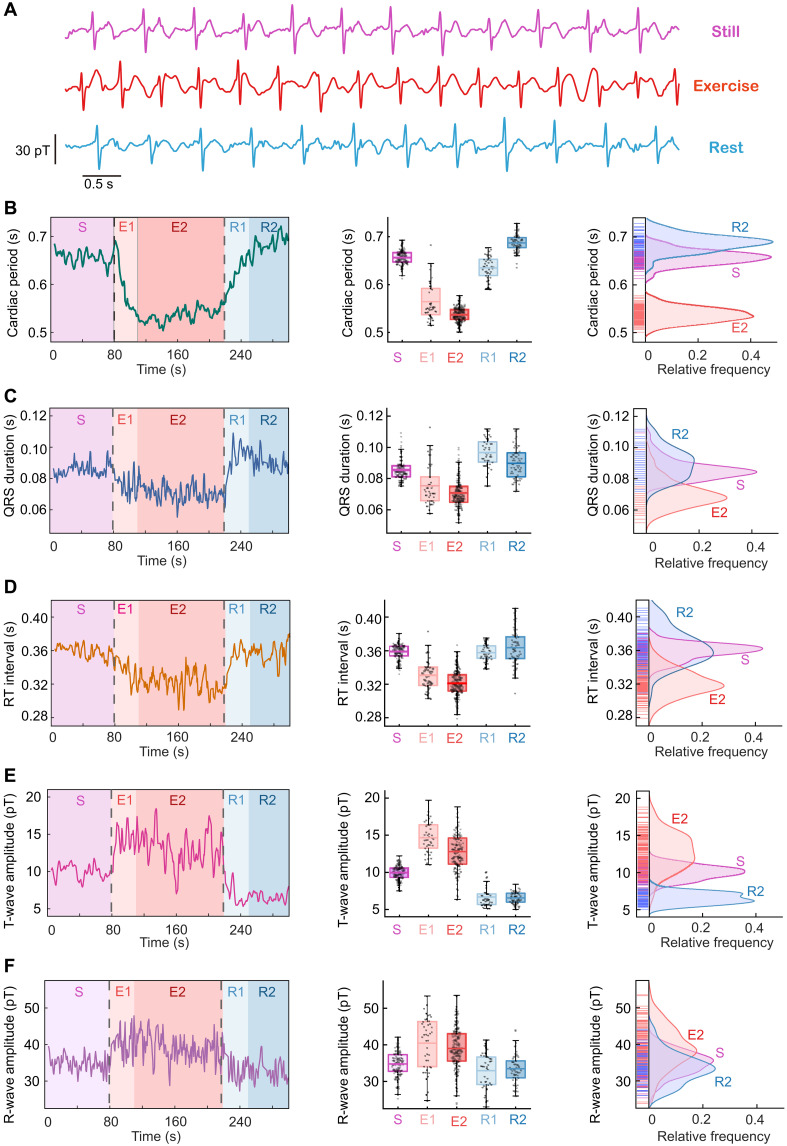
The result of eMCG measurements. (**A**) MCG signals of the participants under the state of standing still (purple line), doing high-knee exercise (red line), and standing rest (blue line). (**B**) Cardiac period (RR interval) in different states. At the beginning of exercise, the heart period gradually decreases in the E1 stage in about 20 s and then becomes stable in the E2 stage. After the stop of exercise, the cardiac period gradually increases in the R1 stage in about 20 s and becomes stable in the R2 stage. (B to **F**) The left diagram of each figure shows the variation of the characteristic indicators (cardiac period, QRS duration, RT interval, T-wave amplitude, and R-wave amplitude) with time and different states. The middle one shows the statistical results of these indicators in each stage, in which each gray point represents an indicator result in a cardiac cycle, the dashed line represents the average, the colored box represents the distribution range of 25 to 75%, and the vertical bar represents the range within 1.5 interquartile range. The right one shows the distribution map of these indicators of the S, E2, and R2 stages. Compared with the S stage and the R2 stage, the participants have a shorter cardiac period, QRS duration and RT duration, and larger amplitude of T-wave and R-wave in the E2 stage of exercise.

Because the eMCG signal is contaminated by the motion artifacts that are almost two orders of magnitudes larger than the eMCG signal, we design a set of denoising methods to process the raw data to extract clear eMCG signals (see details in Materials and Methods). Figure 3A shows the clear eMCG signals that we obtained after signal processing, in which we can distinguish the QRS complex and T-waves in all these three stages. The ECG and PPG sensors fail to work due to the serious disturbance caused by strong vibration during the exercise, and thus, their results are not shown in Fig. 3A.

To quantitatively analyze the features of eMCG signals, especially the exercise stage, we select six characteristic indicators to describe the eMCG signal periods and waveforms: the cardiac period (RR interval), QRS duration, RT interval, T-wave amplitude, and R-wave amplitude. Figure 3 (B to F) shows these six indicators estimated from the eMCG signal in the time domain within the 300-s eMCG test. In the exercise stage, the cardiac activity is not stable during the first 20 s (E1 stage) and becomes stable during the next 120 s (E2 stage). Similarly, in the rest stage, the cardiac activity is not stable during the first 20 s (R1 stage) and becomes stable during the next 60 s (R2 stage).

In Fig. 3, B to F, the left subfigures show the variations of these eMCG characteristic indicators with time and experiment stages. For example, the cardiac period (Fig. 3B) of a participant is about 0.65 s in the S stage and decreases after the beginning of exercise (E1 stage) until a relatively stable value of 0.52 s (E2 stage), indicating a heart rate of 115 beats per minute. After the participant stops exercise, the cardiac period increases gradually (R1 stage) until a relatively stable value of 0.69 s (R2 stage). The middle subfigures show the statistical results of these indicators in each stage, and the right subfigures show the distribution maps of these indicators in S, E2, and R2 stages. From these statistical results and distribution maps, we can find that the SD of the indicators in E2 stage is larger than in the S and R2 stages, which may be caused by the larger deviation of each heartbeat from identity and the stronger noise of eMCG signals in the exercise stage. Besides the cardiac period, we also find that the QRS duration and RT interval slightly decrease in E2 stage, which is reasonable due to the faster heart rate in exercise, as shown in Fig. 3 (C and D).

Furthermore, for the amplitude of eMCG signals, the amplitude of T-wave increases from ∼10 pT in the S stage to ∼15 pT in the E2 stage and decreases to 6 pT in the R2 stage. The amplitude of R-wave increases from ∼35 pT in the S stage to ∼40 pT in the E2 stage and decreases to 33 pT in the R2 stage. The increment of T- and R-wave amplitudes may result from the energetic exercise leading to stronger cardiac activities. Comparing the characteristic indicators before and after exercise, we notice that the cardiac activity in the R2 stage is slightly weaker than that in the S stage. For example, the heart rate in the R2 stage is slower than that in the S stage, and the amplitudes of T- and R-wave in the R2 stage are smaller than that in the S stage, as shown in Fig. 3 (E and F). The reason for this phenomenon may be explained by the temporarily tired heart after energetic exercise.

The eMCG measurement shows increased T- and R-wave amplitudes and decreased cardiac periods in the exercise stage, which is in agreement with previous eECG measurements. These results of eMCG measurement prove that our movable MCG system is capable of detecting the MCG signal of a freely behaving participant in an unshielded environment, even if the participant is doing energetic exercise, which enables the unshielded eMCG measurement a reality.

## DISCUSSION

In this work, we demonstrate that our OPM-based movable unshielded MCG system can measure the cardiac magnetic signal of freely behaving participants and has sufficient sensitivity for various MCG applications. In particular, such a prototype wearable MCG system promotes cardiovascular research and clinical applications, such as eMCG tests and long-term MCG recordings. The OPM based on such a scheme is promising to realize a wearable and movable multichannel MCG system that nearly sets no limits to participants. However, there are several technical issues and challenges that get in the way of reaching this goal, such as the suppression of magnetic gradient noise, the miniaturization of sensors for multichannel operations, and the removal of motion artifacts. With these issues solved, the wearable and movable multichannel unshielded biomagnetic measurement system can also be used for recording brain magnetic fields, i.e., magnetoencephalography (MEG) measurements, whose amplitude is about two orders of magnitude weaker than the cardiac magnetic field. Although, there have been some studies on recording the MEG signal of moving participants in a MSR (*44*) and the unshielded static MEG measurements (*35*, *37*), a MEG system that is both unshielded and movable remains to be developed.

For the unshielded MCG measurements, the magnetic gradient noise is the main factor of limiting the sensitivity of our first-order optically pumped gradiometers. Considering that the first-order gradiometer can only suppress the common-mode magnetic noise, one direct way to suppress the magnetic gradient noise is to operate the OPMs as a second-order gradiometer consisting of more OPM channels (≥3). The second-order gradiometer, made up of two first-order gradiometers, can greatly reduce the magnetic field gradient noise from surroundings (*45*). To develop a second-order gradiometer, the magnetic sensors should be further miniaturized. A low-noise light source with a smaller size, such as the vertical-cavity surface-emitting laser, and a more compact structure are under investigation for miniaturized multichannel OPMs with all-optical modulation constructions.

Compared with resting MCG recordings, the motion artifacts are main factors that should be considered when recording the eMCG signal of participants in motion because the measured magnetic fields during exercise are often heavily contaminated by many kinds of noise. Although, the MCG recording does not suffer from the unstable contact between skin and sensors for its noncontact measurement, the variation of magnetic fields of surroundings in motion is another important source of artifacts due to the inhomogeneous geomagnetic fields. By using additional sensors, such as triaxial accelerometers and gyroscopes, to synchronously monitor the movement, many methods have been proposed to detect and suppress the motion artifacts superimposed on MCG (ECG) signals, such as the adaptive filters (*46*) and the stationary wavelet transform (*47*, *48*).

## MATERIALS AND METHODS

### Self-oscillating cesium magnetometer

**Fig. 4.**
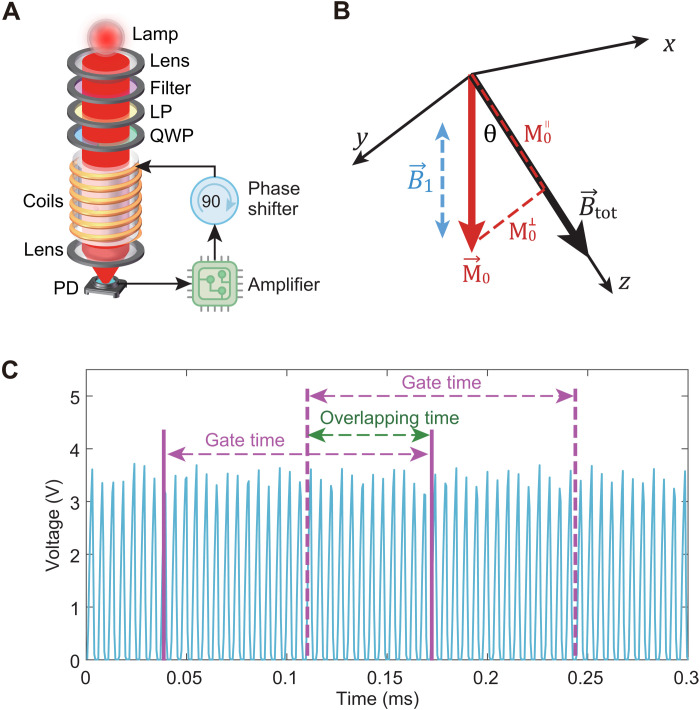
Principle of the self-oscillating magnetometer. (**A**) Schematic of the self-oscillating magnetometer. Broad-spectrum light generated by the cesium lamp is collected and collimated by a lens and sent through an optical band-pass filter to remove the far-detuned light. After passing through these polarization optics (LP and QWP), a circularly polarized light is obtained and used for both optical pumping and probing the atoms confined in the vapor cell. The transmitted light is detected by the photodetector, whose signal is amplified, shifted in phase, and then fed back to drive the magnetic field coil. (**B**) Principle of single-beam self-oscillating magnetometers. Taking the direction of the total magnetic field ${\vec{B}}_{\mathrm{tot}}$ that to be measured as the *z* axis. Without loss of generality, the atomic magnetic moment ${\vec{M}}_{0}$ generated by the pump light is assumed to lie in *yz* plane with an angle of θ with respect to the field ${\vec{B}}_{\mathrm{tot}}$. A radio frequency magnetic field ${\vec{B}}_{1}$ generated by the magnetic field coil is applied along the light to excite the magnetic resonance. Because of the Larmor precession of the atomic magnetic moment around the total magnetic field, only the longitudinal component $M_{0}^{∥}$ parallel to the total magnetic field is preserved and contributes to the magnetic resonance signal. (**C**) Schematic of the Larmor frequency estimation. The output signal is divided into many overlapped segments of equal length where the time duration of each segment is termed as the gate time. The length of the overlap between two adjacent segments is termed as overlapping time, which determines the sampling rate of the magnetic field measurement.

The cesium magnetometer is operated in a self-oscillating mode, where the secular change of the transmitted light intensity is reused as a driving magnetic field to excite the Zeeman resonances. Because of the simple structure, quick response, and no need for lock-in amplifiers or proportional integral derivative feedback systems, it has been well developed and commercialized for many years. Figure 4A shows the schematic diagram of self-oscillating magnetometers. Light from the cesium lamp is collected and collimated by a lens and sent through a linear polarizer and a quarter-wave plate to produce circularly polarized light for both pumping and probing. The light is absorbed by the vapor cell with a pressure of several tens of torr, which results in the magnetic resonance linewidth of 400 Hz, and is then detected with the photodetector. The signal from the photodetector is amplified, shifted in phase, and then fed back to drive the magnetic field coils.

For a scalar magnetometer designed to measure the total magnetic field, there would be a slight dependence of the field measurement on the relative orientation of the sensor to the field (*49*). This unwanted dependence is termed heading error, which is important for magnetic field measurements in moving platforms. To suppress the heading error of the magnetometer, a beam-splitting method is used where the quarter-wave plate is customized to generate two beams with opposite circular polarization. In this case, the heading error caused by the light shift is suppressed. Besides, all the components of the sensor are carefully selected and tested to be nonmagnetic to minimize the heading error caused by the sensor materials. We maintain an angle of 45° between the magnetic field and pumping light directions, thereby optimizing the sensitivity of magnetometers. Normally, the magnetometer oscillates spontaneously at the Larmor frequency if the loop gain is unity and the loop phase shift is zero (*50*). The vapor cell, including all the optical components, such as the lens, wave plates, magnetic field coils, and the photodiode, is enclosed in a homemade structure, which has a size of 64 mm in diameter and 160 mm in height.

To achieve a high CMRR of the OPMs, the responses of the two OPMs with respect to the same magnetic field signal should be as close as possible. If we want to realize a high CMRR in a low-frequency band for MCG signals, then the OPMs should have wide bandwidths, which usually benefit the consistency of OPMs (*51*). Considering that the OPM we developed is based on a self-oscillating scheme and its output is a series of square waves at the Larmor frequency that is proportional to the magnetic field strength, we need to estimate the frequency to obtain the information of the magnetic field. A frequency estimation program to precisely estimate the measured magnetic field of the OPMs is developed (see details in Materials and Methods). With the frequency estimation program, we obtain a wide bandwidth of OPMs without reducing the magnetic sensitivity. The −3-dB bandwidths of the two OPMs are both ∼180 Hz, which is primarily determined by the parameter settings of the frequency estimation program, especially the gate time. For a shorter gate time, the bandwidths of OPMs can reach several kilohertz or even higher at the cost of poorer sensitivities, because the shorter gate time indicates a lower frequency resolution when analyzing the data with Fourier transform.

### Larmor frequency estimation

As mentioned above, the output of the self-oscillating magnetometer is a series of square waves whose frequency is proportional to the strength of the magnetic field. Therefore, measuring the frequency of the repetitive signal in a low-noise floor is an important part of obtaining a high-sensitivity magnetic field measurement. For the application of frequency measurements, the electronic instrument called a frequency counter is commonly used. Most frequency counters work by using a counter which accumulates the number of events occurring within a specific period of time. After a preset period known as the gate time, the frequency is obtained by dividing the number of detected events by the gate time and then the counter is reset to zero for the next measurement. In this case, the frequency counter usually has a limited sampling rate due to the gate time, and the limited sampling rate indicates a narrow bandwidth of the magnetometer. Although the sampling rate can be increased by setting a shorter gate time, it usually results in a higher-noise floor of the frequency measurement due to a lower frequency resolution.

Therefore, we have developed a custom frequency estimator program rather than using the frequency counter to measure the frequency. With a high-speed data acquisition card, we sample the analog repetitive signal at a sampling rate of 2 MHz. The collected data are processed with the frequency estimator program with a gate time of 10 ms and an overlap time of 9 ms, as shown in Fig. 4B. In this case, the sampling time is 1 ms, the difference between the gate time and the overlap time. With a frequency estimation algorithm that is based on discrete Fourier transform, we can measure the frequency both in a high sampling rate of 1 kHz and a low-noise floor.

### Measuring MCG signal with scalar magnetometers

**Fig. 5.**
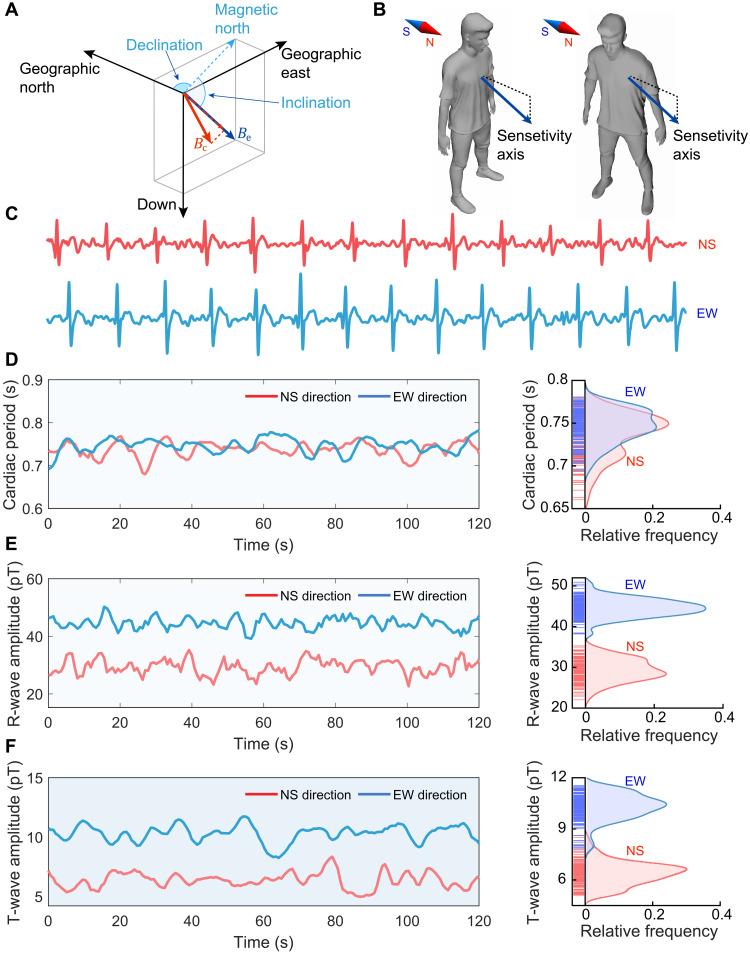
MCG signals in different directions. (**A**) Superposition of the background field (Earth’s field *B*_e_) and the cardiac magnetic field *B*_c_. (**B**) Schematic of the orientation of participants in respect to the Earth’s field. (**C**) Measured MCG signal in the north-south (NS) direction and the east-west (EW) direction. (**D**) Measured cardiac period within the measurement time of 120 s. (**E** and **F**) Measured amplitude of R-wave and T-wave. The MCG signal in the EW direction shows a larger amplitude than that in the NS direction.

As a magnetic resonance–based OPM, which performs scalar measurements of magnetic fields, we obtain the total magnitude of the magnetic field independent of orientation. In this case, the measured MCG signal *B*_c_ is extracted from the variation of the total field. Under the bias Earth’s field *B*_e_ ≈ 54,000 nT that is many orders of magnitude larger than the MCG signal to be measured, the total magnetic field recorded by the OPM can be formulated as

$$B_{\mathrm{tot}}=\sqrt{{\left(B_{e}+B_{c,l}\right)}^{2}+B_{c,t}^{2}}$$ (1A)

$$\approx B_{e}+B_{c,l}+\frac{B_{c,t}^{2}}{2(B_{c,l}+B_{e})}$$ (1B)

where *B*_c,l_ and *B*_c,t_ are the longitudinal and transverse components of the MCG signal *B*_c_, respectively, with respect to the Earth’s field *B*_e_. Because the Earth’s field *B*_e_ ≫ *B*_c_, Eq. 1A indicates that almost only the longitudinal component of MCG signals contributed to the total field and the OPM measurement results are insensitive to the transverse component of MCG signals, as shown in Fig. 5A. As a result, the scalar OPM actually performs a single-axis vector measurement, instead of a scalar measurement, of the weak cardiac field under the Earth’s field. The single-axis vector measurement suggests that we can sample different components of the cardiac field by changing the orientation of participants with respect to the Earth’s field.

Therefore, for a given biomagnetic signal, the measurement results of scalar OPMs are related to the orientation of participants with respect to the background field (*52*). For the experimental place, the magnetic inclination is 59.4° and the magnetic declination is −7.3°. To demonstrate the characteristics of MCG signals in different directions, we conducted two experiments as shown in Fig. 5B, in which the participants stand on the ground and face north or east. Figure 5 (C to F) shows the measurement results of MCG signals in different directions. Because the MCG signal is relevant to cardiac activities, the MCG signal is recorded after participants stand on the ground and rest for several minutes for avoiding the exercise or other factors that may affect cardiac activities. Figure 5D shows that the participants in the two cases, facing north and facing east, have similar cardiac periods, which indicates the similar intensity of cardiac activities. Although, the measured MCG signal in two directions have similar waveforms, the measurement results shown in Fig. 5 (E and F) suggest that the recorded MCG signal is stronger with larger amplitudes of R-wave and T-wave when the participant is standing facing east. Therefore, for practical MCG signal recordings based on the scalar OPM, an auxiliary three-axis fluxgate sensor will be needed for signal calibrations.

### Signal processing

After MCG signals are acquired with OPMs, the signal processing is carried out to increase the SNR of the MCG signals. The raw MCG signal is typically contaminated by various types of artifacts and noise during its acquisition, such as the high-frequency noise (magnetomyogram-induced noise, additive white Gaussian noise, and power line interference) and the low-frequency noise (baseline drift and respiratory motion). For our experiments with movable OPMs, the artifact produced by the movement in Earth’s field should also be considered. Therefore, to obtain a clear MCG signal, the signal denoising is required. Many denoising methods have been reported to suppress the noise of ECG signals, such as filter banks, principal components analysis (*53*), independent component analysis (*54*, *55*), empirical mode decomposition (EMD) (*56*–*58*), and wavelet transform (*59*). Because both the ECG signals and MCG signals are produced by heart activities and feature a similar waveform, the denoising methods used in ECG can also be applied in MCG signal denoising.

**Fig. 6.**
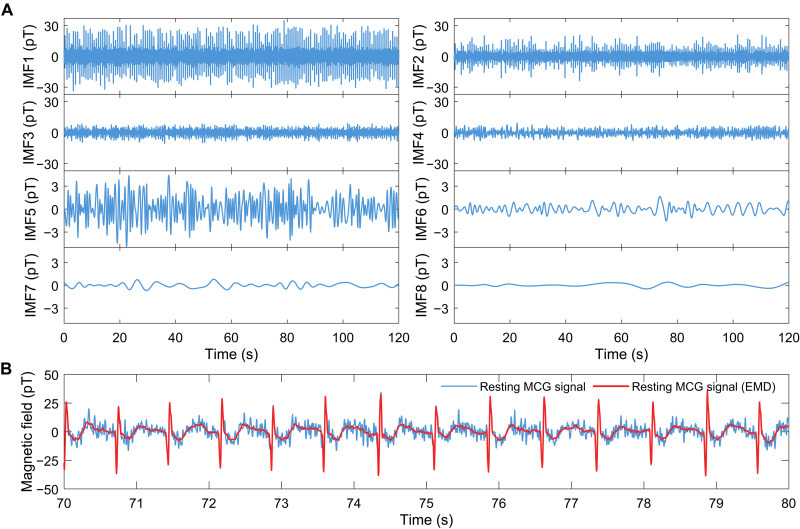
MCG signal denoising based on EMD. (**A**) The resting MCG signal is decomposed into different intrinsic mode functions (IMFs) based on local characteristics of it in time scale. (**B**) The resting MCG signal before (blue) and after (red) EMD-based denoising. The reconstructed MCG signal is cleaner and easier to analyze.

The raw measured MCG signals are first filtered with a fourth-order 0.6- to 45-Hz Butterworth band-pass filter to remove the baseline drift and other high-frequency noise. Besides, to remove the powerline interference and other frequency spikes caused by the cross-talk of OPMs and surrounding noise, as shown in Fig. 1B, the fourth-order notch filters are also applied to denoise the MCG signals. As the resting MCG signal, for example, the blue line in Fig. 6B shows the filtered resting MCG signals, where the QRS complex is clearly observed, while the P-wave cannot be distinguished easily. To further reduce the noise of MCG signal, the EMD method is used. The EMD is a data-driven method for analyzing time series signals by decomposing the signal *s*(*t*) into a number *L* of so-called intrinsic mode functions (IMFs)

$$s(t)=\sum_{i=1}^{L}{\mathrm{IMF}}^{\left(i\right)}(t)+{\mathrm{Res}}_{L}(t)$$ (2)

where IMF^(*i*)^(*t*) is the *i*th IMF component of the signal *s*(*t*) and Res_L_(*t*) is the residual signal that contains long-period variations. The extracted modes IMF^(*i*)^(*t*), which have different time scales, are nearly orthogonal to each other. Compared to other analysis methods like Fourier transforms and wavelet decomposition, the EMD is suitable for analyzing natural signals, which are usually nonstationary and nonlinear. Figure 6A shows the IMFs and residual components of the resting MCG signal based on the EMD. The number of IMFs is determined by the bandwidth of the OPM to separate the data in different time scales. To locally suppress low-energy IMF parts that are considered to be contaminated by the noise, the EMD interval thresholding (EMD-IT) denoising method is used (*60*). Different from the traditional EMD direct thresholding, where only one threshold is selected for every IMF, EMD-IT considers the zero-crossing interval as a whole to perform thresholding. The absolute value of the extreme point is taken as the judgment standard to decide whether the unit is considered to be reserved or eliminated. In this case, EMD-IT is more suitable for the case where the IMF component crosses zero intermittently and can effectively reduce the discontinuity of the reconstructed signal. According to the decomposed IMFs, the corresponding evaluation parameters are calculated, threshold processing is carried out, and the denoised resting MCG signal is reconstructed. As shown in Fig. 6B, the residual noise contained in the resting MCG signal is effectively removed and the basic features of the MCG waveform are retained, which ensures that the conclusions that we derived from the denoised MCG signals are real rather than a fake phenomenon caused by unsuitable data processing.

## Notes

View/request a protocol for this paper from *Bio-protocol*.
