# Supplementary material for: A movable unshielded magnetocardiography system
Source: Sci Adv. 2023 Mar 29;9(13):eadg1746. doi: 10.1126/sciadv.adg1746 (PMC10058232; doi:10.1126/sciadv.adg1746)
Supplement: Supplementary file 1 — Supplementary Text Figs. S1 and S2 [file sciadv.adg1746_sm.pdf]

Science Advances
AAAS

Supplementary Materials for

A movable unshielded magnetocardiography system

Wei Xiao *et al.*

Corresponding author: Teng Wu, wuteng@pku.edu.cn;
Hong Guo, hongguo@pku.edu.cn

Sci. Adv. **9**, eadg1746 (2023)
DOI: 10.1126/sciadv.adg1746

This PDF file includes:

Supplementary Text
Figs. S1 and S2

Supplementary Text

Section S1: Single-beam self-oscillating magnetometer

The self-oscillating magnetometer (SOM) is a high-sensitivity magnetometer based on magnetic resonances. It features a simpler structure without lock-in amplifiers or PID (Proportion Integration Differentiation) controllers and a fast response to the variation of magnetic field compared to the non-self-oscillating scheme. As a much simple Mx magnetometer, the radio-frequency (rf) magnetic field that excites the magnetic resonance is generated by the self-oscillation of the atomic ensemble itself. A single-beam arrangement is employed to further simplify the structure of magnetometers.

As Fig. 4 shown, the main elements are almost the same as a typical Mx or bell-bloom magnetometer, which includes a resonant light source, optical elements, an atomic cell, a photodetector, and electronic circuits. The light source for the SOM is a cesium spectral lamp that contains atoms of the same type as in the atomic cell. The cesium lamp is placed in a microwave field, causing an electronic discharge accompanied by the emission of resonant light. The resonant light is sent through a lens and quarter-wave plates to make a collimated and circularly polarized light. The light is used both for optical pumping of the cesium atoms in the atomic cell filled with buffer gas and for resonance detection. The intensity of the transmitted light passing through the cell is detected by a photodiode (PD) and then sent to the electronic circuits. The electronic circuits mainly contain two parts, the amplifier, and the phase-shifter. The amplifier is used for extracting and amplifying the Larmor precession signal from the original PD signal. The phase-shifter is used for adding a 90 degrees phase shift to the signal from the amplifier, because of the intrinsic phase shift between the rf field and the probe-beam signal. The signal after being processed with the electronic circuits is again transferred to the cesium atoms by magnetic field coils to excite the magnetic resonances. In this case, the SOM works as an atomic frequency generator where atoms will oscillate spontaneously at the Larmor frequency if the loop gain is unity with zero phase shift. An angle of 54.7 degrees between the external bias field and light can optimize the magnetometer to a better sensitivity.

We use the following Bloch equation,

$$\frac{\mathrm{d}}{\mathrm{d}t}\mathbf{M} = \gamma\mathbf{M} \times \mathbf{B} - \Gamma_0\mathbf{M} + R_{\mathrm{OP}}(M_0\hat{z} - \mathbf{M}), \tag{S1}$$

to describe the mechanism of the single-beam SOM. The $\mathbf{M} = M_x\hat{x} + M_y\hat{y} + M_z\hat{z}$ is the atomic magnetic moment, in which M_x, M_y and M_z are the projections of the magnetic moment along three directions. The first term on the right-hand side of Eq. (S1) represents the Larmor precession of $\mathbf{M}$ along the magnetic field $\mathbf{B}$, where γ is the gyromagnetic ratio of atoms. The second term describes the spin relaxation due to various collisions and other relaxation factors. The last term describes the optical pumping at a rate R_{OP}, in which $M_0\hat{z}$ is the atomic magnetic moment created by a z−axis pumping light without any magnetic fields and relaxation.

Considering a general case where there is an angle θ between the pumping light and the bias field, as shown in Fig. 4B. In this case, only the magnetic moment along the bias field $\mathbf{B}_0$ is reserved and the magnetic moment perpendicular to the bias field is averaged to zero due to the Larmor precession. Without loss of generality, we assume the bias field and the pumping light are in *y*-*z* plane. To excite the magnetic resonance, a weak rf field $B_1\cos\omega t$ is applied along the pumping light. By decomposing the oscillating field into two counter-propagating rotating fields, such that

$$B_1 \cos\omega t = \frac{B_1}{2}\left(e^{-i\omega t} + e^{i\omega t}\right), \tag{S2}$$

we can solve Eq. (S1) in the co-rotating reference frame. With further applying the rotating wave approximation, the spin response to the driving rf field along the pumping light can be approximately expressed as

$$M_{\mathrm{pump}} \approx \frac{1}{2} M_0 \gamma B_1 \sin^2\theta \cos\theta \frac{\Gamma \sin\omega t + (\omega - \gamma B_0)\cos\omega t}{\Gamma^2 + (\omega - \gamma B_0)^2}, \tag{S3}$$

where $\Gamma = \Gamma_0 + R_{\mathrm{OP}}$ is the total spin relaxation rate. We can easily find that the angle of $\theta = \arctan\sqrt{2} \approx 54.7$ degrees can maximize the amplitude of the spin response along the pumping light. So, to achieve the optimal performance of the magnetometer, we should try to maintain an angle of 54.7 degrees between the light and the bias field. Besides, Eq. (S3) also indicates that there is a 90 degrees phase shift between the spin response signal and the exciting rf field. So, the single-beam SOM needs a phase shifter to add a 90 degrees phase shift to the PD signal.

Section S2: Frequency estimation algorithm

Since the output of the SOM is a series of square waves at the Larmor frequency that is proportional to the magnetic field strength, we need a method to read the self-oscillation frequency to obtain the information of the magnetic field. In this case, the performance of the SOM also depends on the method of frequency estimation. Typically, a device called a frequency counter is commonly used for measuring the Larmor frequency. Most frequency counters work by using a counter to accumulate the number of events occurring within a specific period of time (called gate time τ_{g}), such as the Keysight 53200 series frequency counter. However, such kind of frequency counters usually has a limited sampling rate f_s, which is the inverse of the gate time ($f_s = 1/\tau_{\mathrm{g}}$). Besides, to obtain a high bandwidth of the SOM, we need to set a shorter gate time in the frequency counter, which often leads to a higher noise floor of the SOM.

To solve the limited sampling rate and poor performance of the frequency counter, we develop a frequency estimation algorithm to measure the Larmor frequency instead of the frequency counter. By acquiring the original signal of the SOM with a high-speed data acquisition card and extracting the Larmor frequency from the original signal with the frequency estimation algorithm, we realize a high bandwidth and high sensitivity magnetometer with little loss of sensitivity in a short gate time, as shown in Fig. S1.

Figure 4 shows the basic principle of the frequency estimation algorithm where there are two characteristic parameters, the gate time τ_{g} and the overlapping time τ_{o}, to determine the bandwidth and the sampling rate of magnetometers. The overlapping time is the overlapped time span between the two adjacent time segments that are used for extracting the Larmor frequency. In this case, the sampling rate is determined by the equation $f_s = 1/(\tau_{\mathrm{g}} - \tau_{\mathrm{o}})$, which can be much higher than that of the frequency counter for the same gate time. A higher sampling rate can not only suppress the aliasing noise but also be helpful for the magnetocardiography (MCG) signal processing.

The frequency estimation of a single sinusoid embedded in noise is one of the classical problems of signal processing. There are many kinds of frequency estimators have been proposed, such as the maximum likelihood estimator, Kay's window estimator, etc. The frequency estimation algorithm we used to estimate the Larmor frequency from every time segment is based on a modified Rife frequency estimation algorithm. The modified Rife frequency estimator is based on the discrete Fourier transform (DFT) of the observations followed by an initial coarse search for the frequency having the greatest magnitude of the spectrum. A finer frequency estimate can be obtained using a method such as the secant method.

Section S3: Magnetic field noise in the open environment

Since the MCG signal is usually much weak than the magnetic field noise of surroundings, the magnetic field noise in an open environment is an important characteristic that should be considered when measuring MCG signals without magnetic shields. To fully characterize the field noise of surroundings, we have measured the magnetic field noise with two magnetometers that are separated at a distance of ∼15 cm. Figure S2 shows the measurement results of the magnetometers for 300 seconds. The slow drift of the environmental field is about 20 nT within the duration of 300 seconds, which is mainly caused by the diurnal variation, working electronic devices, passing pedestrians, cars and even the underground railways. The experimental place is located in a park and the nearest underground railway is about two kilometers away. A high-voltage line that across over the experimental place leads to a strong power-line interference (up to about 15 nT) to the magnetometers, as shown in the inset of Fig. S2A. Figure S2B shows a noise floor of several $pT/Hz^{1/2}$ in the open environment, where the frequency spikes in the noise spectral density are mainly caused by the 50 Hz electric noise. The measured noise floor indicates that the experimental place is relatively magnetic quiet compared to the bustling downtown. However, we expect the magnetometers can work normally and still have a good signal-to-noise ratio (SNR) of measured MCG signals in a noisier environment, according to the measured common-mode noise rejection ratio (CMRR) shown in Fig. 1B.

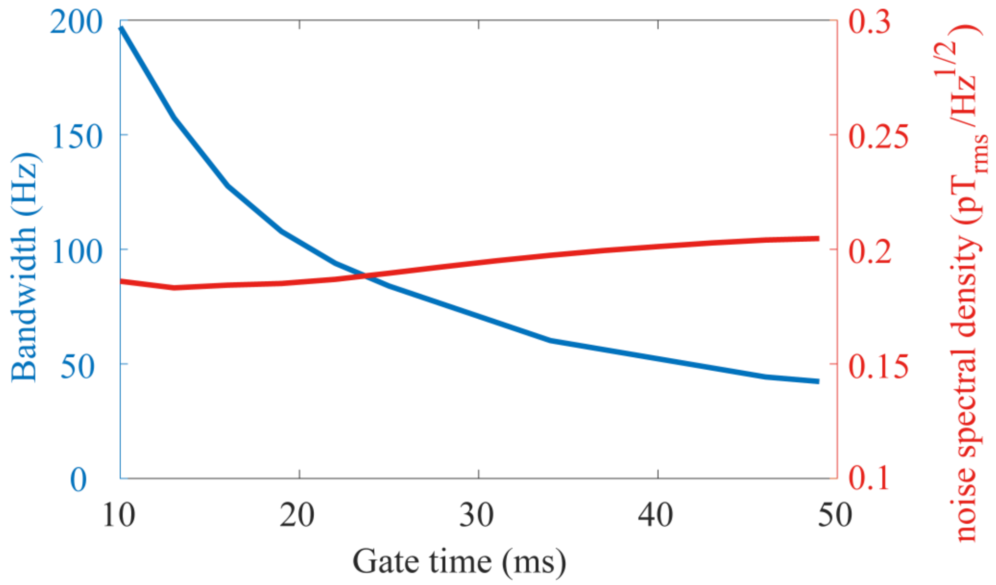

Fig. S1. The dependence of magnetometer performance on gate time. Different gate time leads to different bandwidths (blue line) and sensitivities (red line) of the gradiometer. As the gate time increases, the bandwidth of the magnetometer decreases but its sensitivity only decreases slightly due to the increased inconsistency of the two magnetometers.

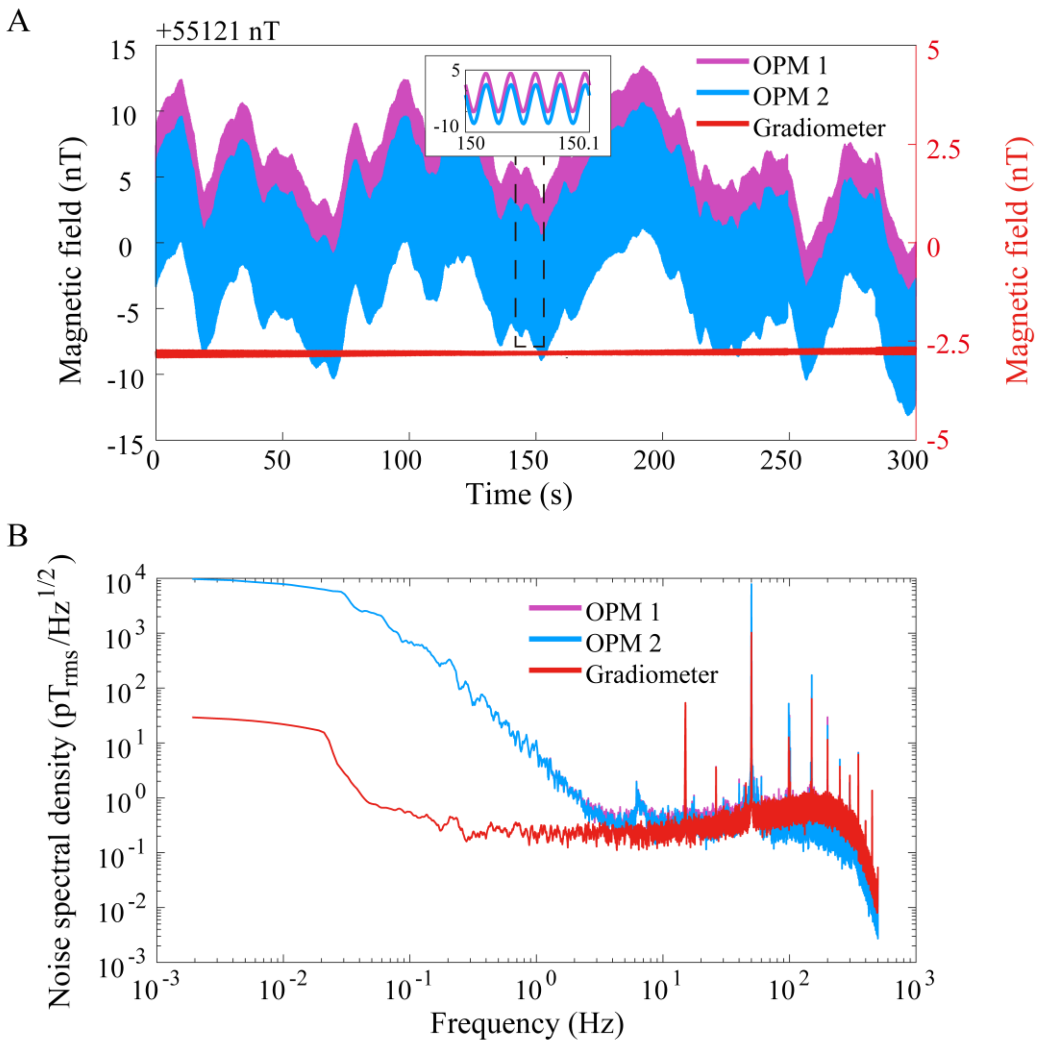

Fig. S2. The magnetic field noise of the open experiment environment. (**A**) the measured magnetic field fluctuations for 300 seconds. The inset shows the field noise ranging from 150 s to 150.1 s. (**B**) the measured magnetic field noise spectral density of the magnetometers.
